# Supplementary material for: Analyzing the proximity to cover in a landscape of fear: a new approach applied to fine-scale habitat use by rabbits facing feral cat predation on Kerguelen archipelago
Source: PeerJ. 2016 Mar 7;4:e1769. doi: 10.7717/peerj.1769 (PMC4793317; doi:10.7717/peerj.1769)

Supplemental information file 1 Blanchard et al.

Photo credit : P. Blanchard

Study area in Pointe Morne (49°22’S, 70°26’E), Kerguelen archipelago


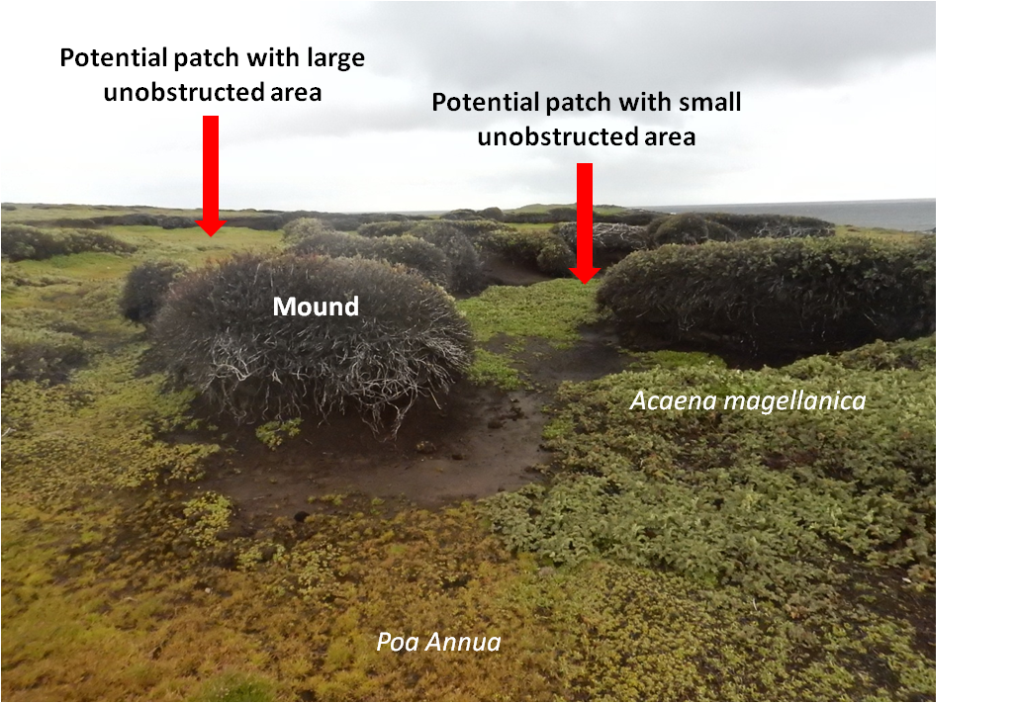

Supplement: File S1 [file peerj-04-1769-s004.docx]
