# Supplementary material for: Analyzing the proximity to cover in a landscape of fear: a new approach applied to fine-scale habitat use by rabbits facing feral cat predation on Kerguelen archipelago
Source: PeerJ. 2016 Mar 7;4:e1769. doi: 10.7717/peerj.1769 (PMC4793317; doi:10.7717/peerj.1769)
Supplement: File S2 [file peerj-04-1769-s005.docx]

Supplemental information file 2 Blanchard et al.

################################ 1. AREAS #############################

# Calculation of the area of each sector. Data are: the radius of the theoretical circle, the two distances between the center of the patch and both extremities of the cover object and the angle (radian) between both extremities of the cover.

surface=function(R,R1,R2,theta)

{

if(is.na(R1)) {R1=100*R} # if the distance to the cover is too large to be measured, it is taken as 100 times larger than the radius of the theoretical circle

if(is.na(R2)) {R2=100*R}

r1=min(R1,R2) # r1 is the small distance to the cover

r2=max(R1,R2) # r2 is the large distance to the cover

if (r2<=R)

{

surf=r1*r2*sin(theta)/2

} else

{

if (r1>=R)

{

aa=atan((r1-r2*cos(theta))/(r2*sin(theta)))

aa=min(aa,theta-aa)

if (r1*r2*abs(sin(theta))/sqrt(r1^2+r2^2-2*r1*r2*cos(theta))<R & aa>0) # evaluates the minimal distance to the cover. Tests whether this distance is larger than the radius of the theoretical circle

{

cosa1=(r1*r2^2*sin(theta)^2+sqrt((R^2*(r1^2+r2^2)-r1^2*r2^2+(r1*r2*cos(theta)-R^2)^2-R^4)*(r2*cos(theta)-r1)^2))/(r1^2+r2^2-2*r1*r2*cos(theta))/R

cosa2=(r1*r2^2*sin(theta)^2-sqrt((R^2*(r1^2+r2^2)-r1^2*r2^2+(r1*r2*cos(theta)-R^2)^2-R^4)*(r2*cos(theta)-r1)^2))/(r1^2+r2^2-2*r1*r2*cos(theta))/R

a1=acos(round(cosa1,14))

a2=acos(round(cosa2,14))

A1=min(a1,a2)

A2=max(a1,a2)

surf1=R^2*A1/2

surf2=R^2*sin(A2-A1)/2

surf3=R^2*(theta-A2)/2

surf=surf1+surf2+surf3

} else

{

surf=R^2*theta/2

}

} else

{

if (r2*cos(theta)<r1)

cosa=(r1*r2^2*sin(theta)^2-sqrt((R^2*(r1^2+r2^2)-r1^2*r2^2+(r1*r2*cos(theta)-R^2)^2-R^4)*(r2*cos(theta)-r1)^2))/(r1^2+r2^2-2*r1*r2*cos(theta))/R else

cosa=(r1*r2^2*sin(theta)^2+sqrt((R^2*(r1^2+r2^2)-r1^2*r2^2+(r1*r2*cos(theta)-R^2)^2-R^4)*(r2*cos(theta)-r1)^2))/(r1^2+r2^2-2*r1*r2*cos(theta))/R

a=acos(cosa)

surf1=R^2*(theta-a)/2

surf2=r1*R*sin(a)/2

surf=surf1+surf2

}

}

return(surf)

}

# Calculation of the overall surface (i.e. of the “domain of safety”)

# Data are: the radius of the theoretical circle, the list of the distances and angles (in radian) of the different cover items

Surface=function(R,dist_begin,angle_begin,dist_end,angle_end)

{

nbre_fragment=length(dist_begin)

S=0

for (i in 1:nbre_fragment)

{

theta=ifelse(angle_end[i]-angle_begin[i]>0,angle_end[i]-angle_begin[i],angle_end[i]-angle_begin[i]+2*pi)

s=surface(R,dist_begin[i],dist_end[i],theta)

S=S+s

}

return(S)

}

################################### 2. PLOTS #############################

# Calculation of the angle. Data are: the two distances to the cover, the angle (radian) and the radius of the theoretical circle

Angles=function(R1,R2,theta,R)

{

if(is.na(R1)) {R1=100*R}

if(is.na(R2)) {R2=100*R}

r1=min(R1,R2)

r2=max(R1,R2)

if (r2<=R)

{

angles=-1

} else

{

if (r1>=R )

{

aa=atan((r1-r2*cos(theta))/(r2*sin(theta)))

aa=min(aa,theta-aa)

if (r1*r2*abs(sin(theta))/sqrt(r1^2+r2^2-2*r1*r2*cos(theta))<R & aa>0)

{

cosa1=(r1*r2^2*sin(theta)^2+sqrt((R^2*(r1^2+r2^2)-r1^2*r2^2+(r1*r2*cos(theta)-R^2)^2-R^4)*(r2*cos(theta)-r1)^2))/(r1^2+r2^2-2*r1*r2*cos(theta))/R

cosa2=(r1*r2^2*sin(theta)^2-sqrt((R^2*(r1^2+r2^2)-r1^2*r2^2+(r1*r2*cos(theta)-R^2)^2-R^4)*(r2*cos(theta)-r1)^2))/(r1^2+r2^2-2*r1*r2*cos(theta))/R

a1=acos(round(cosa1,14))

a2=acos(round(cosa2,14))

A1=min(a1,a2)

A2=max(a1,a2)

angles=c(A1,A2)

} else

{

angles=-2

}

} else

{

if (r2*cos(theta)<r1)

cosa=(r1*r2^2*sin(theta)^2-sqrt((R^2*(r1^2+r2^2)-r1^2*r2^2+(r1*r2*cos(theta)-R^2)^2-R^4)*(r2*cos(theta)-r1)^2))/(r1^2+r2^2-2*r1*r2*cos(theta))/R else

cosa=(r1*r2^2*sin(theta)^2+sqrt((R^2*(r1^2+r2^2)-r1^2*r2^2+(r1*r2*cos(theta)-R^2)^2-R^4)*(r2*cos(theta)-r1)^2))/(r1^2+r2^2-2*r1*r2*cos(theta))/R

a=acos(cosa)

angles=a

}

}

return(angles)

}

# Drawing. Data are: the radius of the theoretical circle, the size of the drawing, the list of the distances and angles (radian) of the different cover objects. Patch has to be described in counterclockwise.

plot_function=function(R,scale,dist_begin,angle_begin,dist_end,angle_end)

{

Data=cbind(dist_begin,angle_begin,dist_end,angle_end)

ang=seq(0,6.3,by=0.01)

nbre_fragment=dim(Data)[1]

X=rep(NA,2*nbre_fragment+1)

Y=rep(NA,2*nbre_fragment+1)

X1=Data[,1]*cos(Data[,2])

X2=Data[,3]*cos(Data[,4])

Y1=Data[,1]*sin(Data[,2])

Y2=Data[,3]*sin(Data[,4])

X[2*(1:nbre_fragment)-1]=X1

X[2*(1:nbre_fragment)]=X2

X[2*nbre_fragment+1]=X[1]

Y[2*(1:nbre_fragment)-1]=Y1

Y[2*(1:nbre_fragment)]=Y2

X[2*nbre_fragment+1]=X[1]

Y[2*nbre_fragment+1]=Y[1]

plot(0,type="n",xlim=c(-scale,scale),ylim=c(-scale,scale),xlab="",ylab="",xaxt="n",yaxt="n")

lines(R*cos(ang),R*sin(ang),col="grey")

for (i in 1:nbre_fragment)

{

R1=Data[i,1]

R2=Data[i,3]

Theta=ifelse(Data[i,4]-Data[i,2]>0,Data[i,4]-Data[i,2],Data[i,4]-Data[i,2]+2*pi)

angles=Angles(R1,R2,Theta,R)

alpha=Data[i,2]

if (length(angles)==1)

{

if (angles==-1)

{

x=c(0,R1*cos(alpha),R2*cos(Theta+alpha),0)

y=c(0,R1*sin(alpha),R2*sin(Theta+alpha),0)

}

if (angles==-2)

{

x=c(0,R*cos(seq(alpha,Theta+alpha,by=0.01)),0)

y=c(0,R*sin(seq(alpha,Theta+alpha,by=0.01)),0)

}

if (angles>=0)

{

if (R1>R2)

{

x=c(0,R*cos(seq(alpha,Theta-angles+alpha,by=0.01)),R2*cos(Theta+alpha),0)

y=c(0,R*sin(seq(alpha,Theta-angles+alpha,by=0.01)),R2*sin(Theta+alpha),0)

} else

{

x=c(0,R1*cos(alpha),R*cos(seq(angles+alpha,Theta+alpha,by=0.01)),0)

y=c(0,R1*sin(alpha),R*sin(seq(angles+alpha,Theta+alpha,by=0.01)),0)

}

}

}

if (length(angles)==2)

if (R1<R2)

{

x=c(0,R*cos(seq(alpha,angles[1]+alpha,by=0.01)),R*cos(seq(angles[2]+alpha,Theta+alpha,by=0.01)),0)

y=c(0,R*sin(seq(alpha,angles[1]+alpha,by=0.01)),R*sin(seq(angles[2]+alpha,Theta+alpha,by=0.01)),0)

} else

{

x=c(0,R*cos(seq(alpha,Theta-angles[2]+alpha,by=0.01)),R*cos(seq(Theta-angles[1]+alpha,Theta+alpha,by=0.01)),0)

y=c(0,R*sin(seq(alpha,Theta-angles[2]+alpha,by=0.01)),R*sin(seq(Theta-angles[1]+alpha,Theta+alpha,by=0.01)),0)

}

polygon(x,y,col="red",border="red")

}

lines(X,Y)

points(0,0,pch=20,cex=3)

}

# end
